# Expression of activated signal transducer and activator of transcription-3 predicts poor prognosis in cervical squamous-cell carcinoma

**DOI:** 10.1038/sj.bjc.6605212

**Published:** 2009-07-28

**Authors:** S Takemoto, K Ushijima, K Kawano, T Yamaguchi, A Terada, N Fujiyoshi, S Nishio, N Tsuda, M Ijichi, T Kakuma, M Kage, D Hori, T Kamura

**Affiliations:** 1 Department of Obstetrics and Gynecology, Kurume University School of Medicine, Fukuoka, Japan; 2 Department of Pathology, Kurume University School of Medicine, Fukuoka, Japan; 3 Biostatistics Center, Kurume University School of Medicine, Fukuoka, Japan

**Keywords:** cervical cancer, signal transducer and activator of transcription-3, immunohistochemical study, squamous-cell carcinoma

## Abstract

**Background:**

Stat3 is a member of the Janus-activated kinase/STAT signalling pathway. It normally resides in the cytoplasm and can be activated through phosphorylation. Activated Stat3 (p-Stat3) translocates to the nucleus to activate the transcription of several molecules involved in cell survival and proliferation. The constitutive activation of Stat3 has been shown in various types of malignancies, and its expression has been reported to indicate a poor prognosis. However, the correlation between the constitutive activation of Stat3 and the prognosis of cervical cancer patients has not been reported.

**Methods:**

The immunohistochemical analysis of p-Stat3 expression was performed on tissues from 125 cervical squamous-cell carcinoma patients who underwent extended hysterectomy and pelvic lymphadenectomy, and the association of p-Stat3 expression with several clinicopathological factors and survival was investigated.

**Results:**

Positive p-Stat3 expression was observed in 71 of 125 (56.8%) cases and was significantly correlated with lymph node metastasis, lymph vascular space invasion, and large tumour diameter (>4 cm) by Fisher's exact test. Kaplan–Meier survival analysis showed that p-Stat3 expression was statistically indicative of a poor prognosis for overall survival (*P*=0.006) and disease-free survival (*P*=0.010) by log-rank test.

**Conclusion:**

These data showed that p-Stat3 expression in cervical cancer acts as a predictor of poor prognosis.

Cervical cancer is the second most common malignant tumour among women worldwide, with an estimated half million new cases per year (Parkin *et al*, 2005). An aetiologic relationship between high-risk human papillomavirus and cervical cancer has been firmly established (zur Hausen, 2002), although the molecular mechanism behind the progression or metastasis of this tumour remains unclear. In recent years, major advances in understanding the molecular basis of various types of cancer has helped bring about the development of novel anticancer therapies. Consequently, much interest has been focused on the signal transducer and activator of transcription 3 (Stat3), a member of the Janus-activated kinase/STAT signalling pathway (Levy and Darnell, 2002).

Signal transducer and activator of transcription 3 was first identified as a DNA-binding factor that selectively binds to the interleukin 6 (IL-6)-responsive element in the promoter of acute phase genes from IL-6-stimulated hepatocytes (Wegenka *et al*, 1993). The Stat3 normally resides in the cytoplasm and can be activated through phosphorylation by cytokines, growth factors, and oncogenic proteins, such as IL-6, epidermal growth factor, Ras, and Src (Zhong *et al*, 1994; Yu *et al*, 1995; Levy and Darnell, 2002; Yu and Jove, 2004). Activated Stat3 (p-Stat3) dimerises and translocates to the nucleus, where its occupation of specific DNA-binding sites results in the increased transcription of several molecules that are involved directly in cell survival and proliferation (Huang, 2007).

Constitutive activation of Stat3 has been shown in various types of malignancies (Watson and Miller, 1995; Gouilleux-Gruart *et al*, 1996; Huang *et al*, 2000; Song and Grandis, 2000; Campbell *et al*, 2001; Scholz *et al*, 2003), and immunohistochemical expression of p-Stat3 has been recognised as a predictor of poor survival. In cervical cancer, however, little is known about the prognostic relevance of p-Stat3, although Chen *et al* (2007) showed the expression of p-Stat3 in an immunohistochemical study. Therefore, the aim of this study was to investigate whether the immunohistochemical expression of p-Stat3 can predict a poor prognosis in patients with cervical squamous-cell carcinoma.

## Materials and methods

### Human tissue specimens and patient information

Tumour tissue was collected from 125 cervical cancer patients with invasive squamous-cell carcinoma who had undergone extended hysterectomy with pelvic lymphadectomy at Kurume University Hospital between 1996 and 2005. Normal cervical tissue was also collected from five removed uteri without malignancy, which served as a control. We studied the follow-up information through May 2007 from patient medical records, with a median follow-up period of 50 months. Histopathological diagnosis was based on World Health Organization classifications, and clinical staging was made according to the International Federation of Gynecology and Obstetrics system. Postoperatively, adjuvant treatment was given in the case of lymph node metastasis, lymph vascular space invasion (LVSI), large tumour diameter (>4 cm), deep stromal invasion (>4/5), invasion into the parametrium, and positive status for surgical margin. The use of tissue blocks and chart review was approved by the Institutional Review Board of Kurume University Hospital.

### Immunohistochemical analysis

Sections (3 *μ*m thick) of formalin-fixed, paraffin-embedded tumour specimens were deparaffinised in xylene and rehydrated in graded alcohol. Antigen retrieval was carried out in Tris/EDTA buffer (Dako Target Retrieval Solution, pH 9.0, DAKO, Kyoto, Japan) by heating in a microwave oven for 30 min at 99°C. The specimens were incubated at 4°C overnight in a 1 : 25 dilution of goat affinity-purified polyclonal antibody against p-Stat3 (Tyr-705, sc-7993, Santa Cruz Biotechnology, Santa Cruz, CA, USA). Slides were washed three times in phosphate-buffer solution and further incubated with a biotinylated secondary antibody for 30 min at room temperature. Next, the slides were rinsed with phosphate-buffer solution and incubated for 5 min with diaminobenzidine. The sections were washed twice with distilled water, counterstained with haematoxylin for 1 min, and washed once each with distilled water and phosphate-buffer solution. Afterwards, the slides were mounted and examined using a bright-field microscope. Two investigators independently evaluated and interpreted the results of immunostaining without knowledge of the clinical data for each patient. In our condition, the positive rate of nuclear staining was less than 10% in most cases and a significant difference was not observed with regard to the intensity of staining. Therefore, the existence of nuclear staining was considered positive immunoexpression. We further categorised the positive group into two subgroups for a detailed analysis of lymph node metastatic patients. Local staining up to 5% of the tumour area was defined as +, and staining over 5% of the area was defined as ++, respectively.

### Transfection of Stat3 small interfering RNA

Pre-designed siRNA (small interfering RNA) Reagent ON-TARGET plus against STAT3 and siSTABLE Non-Targeting siRNA no. 1 were purchased from Dharmacon (Lafayette, CO, USA). Two sets of siRNA were transfected into the cervical cancer cell line, SKG II cells, with lipofectoAMINE 2000 (Invitrogen, Carlsbad, CA, USA) according to the manufacturer's protocol. Cells were allowed to proliferate in wells for 48 h.

### Western blotting

Cells were collected and lysed by LDS sample buffer (Invitrogen). Protein concentrations were quantitated using the DC protein assay kit (Bio-Rad, Hercules, CA, USA) according to the manufacturer's protocol. Equal amounts of cellular proteins were electrophoretically fractionated in 10% PAGE gels, transferred to nitrocellulose membranes, and subjected to immunoblot analysis with specific antibodies against p-Stat3 (Cell Signaling Technology, Beverly, MA, USA) and Bcl-xL, vascular endothelial growth factor (VEGF) (Santa Cruz Biotechnology), and *β*-actin (Sigma-Aldrich, St Louis, MO, USA). Specific bands were visualised using the chemiluminescence reagent kit (Amersham ECL and ECL advance Western Blotting Analysis System; GE Healthcare, Chalfont St Giles, UK). Specific bands were quantified by scanning densitometry using the Image J1.34 software (NIH, Bethesda, MD, USA) version 1.61. The expression status of each sample was standardised by the expression status of *β*-actin.

### Statistical analysis

Statistical analysis was performed using the SAS statistical package version 9.1.3 (SAS Institute Inc., Cary, NC, USA). Survival curves were generated using the Kaplan–Meier method, and comparisons between survival curves were made using a log-rank test. Fisher's exact test was used to analyse discrete variables, and the Jonckheere–Terpstra test was used to analyse the association between the number of lymph nodes and the intensity of p-Stat3 immunoexpression. Multivariate survival analysis was conducted using the Cox proportional hazard method. Only *P*-values less than 0.05 were considered statistically significant.

## Results

### Patient characteristics

The 5-year overall and disease-free survival rates of all patients were 86.1 and 83.1%, respectively. Patient characteristics were presented in Table 1. Significantly low 5-year overall survival rates were seen in patients with the factors of FIGO stage II, large tumour diameter (>4 cm), deep stromal invasion (>4/5), LVSI, parametrial invasion, and lymph node metastasis (log-rank test, data not shown). Significantly low rates of 5-year disease-free survival were shown in patients with factors such as large tumour diameter (>4 cm), deep stromal invasion (>4/5), LVSI, parametrial invasion, and lymph node metastasis (log-rank test, data not shown).

### Expression of p-Stat3 in cervical cancer tissue and normal cervical tissue

**Figure 1:**
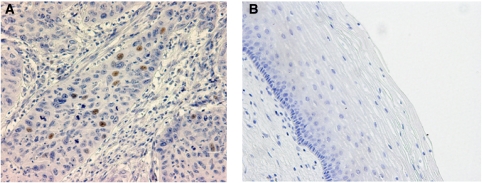
Immunoexpression of p-Stat3 in cervical cancer specimen (**A**), and normal cervical tissue (**B**). Only tumour cell nuclei were stained.

Phosphorylated-Stat3 was expressed only in the nuclei of the tumour cells (Figure 1A) and was not expressed in the nuclei or cytoplasm of normal cells (Figure 1B). Nuclear staining of p-Stat3 was observed in less than 10% of cells in most cases. It was impossible to evaluate the intensity of p-Stat3 staining because no significant difference of intensity status was observed. Therefore, we defined all cases that show nuclear staining of p-Stat3 as positive. No normal cervical tissues were stained with p-Stat3.

### Correlation between clinicopathologic factors and p-Stat3 expression

The correlation between clinicopathological features and positive expression of p-Stat3 is summarised in Table 2. Positive expression of p-Stat3 was significantly associated with large tumour diameter (>4 cm), LVSI, and lymph node metastasis.

### Correlation between lymph node metastases and p-Stat3 expression

**Figure 2:**
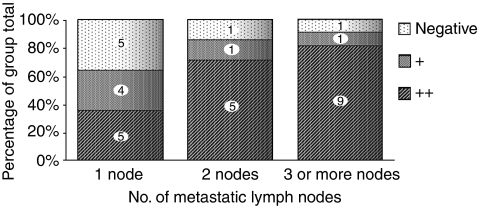
Correlation between the number of metastatic lymph nodes and p-Stat3 expression. The percentages of ‘++,’ ‘+,’ and negative p-Stat3 expression in each group are plotted.

Of the 32 patients with lymph node metastasis, 14 had one node, 7 had two nodes, and 11 had three or more nodes with metastasis. The percentage of p-Stat3 expression status in each group is shown in Figure 2. The relationship between p-Stat3 expression and these three groups was significant, accounting for ordered categories on both factors (*P*=0.022; Jonckheere–Terpstra test).

### Prognostic significance of p-Stat3 expression

**Figure 3:**
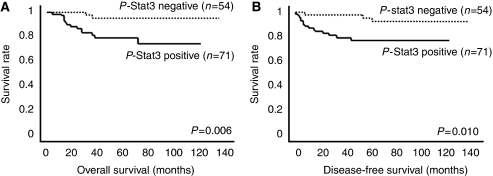
Kaplan–Meier analysis of overall survival (**A**) and disease-free survival (**B**) after extended hysterectomy for p-Stat3-positive or p-Stat3-negative immunostaining.

During the follow-up on 125 patients (with a median follow-up of 50 months), 18 patients (14.4%) developed recurrence and 16 patients (12.8%) died of disease. Among the 125 tumours, 54 tumours (43.2%) were negative and 71 (56.8%) were positive for p-Stat3 expression. The overall and disease-free survival rates were determined using the Kaplan–Meier method. The 5-year overall survival rate for patients with positive expression of p-Stat3 and negative expression of p-Stat3 was 79.2 and 95.3%, respectively. The overall survival rate was significantly higher in patients with p-Stat3-negative tumours than in those with p-Stat3-positive tumours (*P*=0.006; log-rank test; Figure 3A). Similarly, the 5-year disease-free survival rate for patients with positive p-Stat3 expression and negative p-Stat3 expression was 76.8 and 92.3%, respectively, and the disease-free survival rate in patients with negative tumours was significantly higher than that in patients with positive tumours (*P*=0.010; log-rank test; Figure 3B). However, positive p-Stat3 expression was not an independent prognostic factor in multivariate analysis for overall and disease-free survival (data not shown).

Eighteen of the 125 patients in our study developed recurrence during the follow-up period. Of these, 12 had recurrence at a distant site and 6 had local recurrence. Positive expression of p-Stat3 was found in 10 (83.3%) tumours of distant recurrence and 5 (83.3%) tumours of local recurrence. There was no correlation between p-Stat3 expression and the pattern of recurrence.

### Expression of Bcl-xL and VEGF was reduced by inhibiting the expression of Stat3

**Figure 4:**
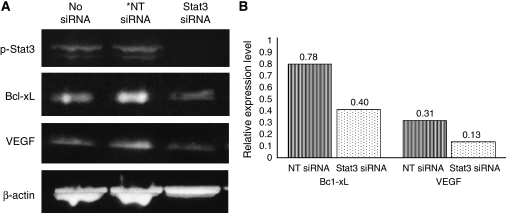
Expression of Bcl-xL, VEGF, and p-Stat3 in cervical cancer cell line and the effect of Stat3 siRNA (**A**). Relative expression level standardised by *β*-actin expression level is indicated (**B**). The expression levels of both Bcl-xL and VEGF with Stat3 siRNA were more reduced than that with No Targeting siRNA (NT siRNA).

Expression of p-Stat3 in all five cervical cancer cell lines was confirmed by western blotting analysis (data not shown). Transfection of siRNA against Stat3 into cervical cancer cell line, SKG II, induced the inhibition of expression of pStat3. Furthermore, the expression of Bcl-xL and VEGF, which are reported to be downstream molecules of Stat3, was decreased (Figure 4).

## Discussion

In this study, we found that Stat3 was activated in 56.8% of cervical squamous-cell carcinomas in immunohistochemical analysis and the expression of activated Stat3 (p-Stat3) significantly correlated with a worse prognosis in patients with this disease by univariate analysis. Immunoexpression of p-Stat3 has been recognised as a predictor of poor survival in other malignancies (Horiguchi *et al*, 2002; Masuda *et al*, 2002; Rosen *et al*, 2006; Yang *et al*, 2007), but, to our knowledge, the correlation between the p-Stat3 expression and prognosis in patients with cervical cancer has not been reported. It has been shown that p-Stat3 immunoexpression was significantly associated with lymph node metastasis or tumour size in other malignancies (Masuda *et al*, 2002; Kusaba *et al*, 2005; Suiqing *et al*, 2005; Shah *et al*, 2006). Some clinicopathological parameters such as FIGO stage, tumour size, deep stromal invasion, LVSI, parametrium invasion, lymph node metastasis, and the number of metastatic lymph nodes have been reported as predictors of poor prognosis in cervical cancer (Sakuragi *et al*, 1999; Waggoner, 2003; Kim *et al*, 2005). Therefore, we examined the possible correlation between such parameters and p-Stat3 expression in cervical cancer specimens, and found that p-Stat3 expression significantly correlated with lymph node metastasis, LVSI, and large tumour size (>4 cm) in these patients. These factors might contribute to a worse prognosis in patients with Stat3 activation. On the other hand, no independent prognostic factor for overall survival was identified by multivariate analysis. Deep stromal invasion (>4/5) was the only independent prognostic factor for disease-free survival. Although p-Stat3 expression was not an independent prognostic factor for either overall survival or progression-free survival, probably because of the correlation with other clinicopathological factors in our study, it was interesting to observe that the hazard ratio was the highest in the significant prognostic factor by univariate analysis (data not shown). This result indicated that p-Stat3 expression can be an important factor to predict prognosis.

Only one immunohistochemical study has been reported on p-Stat3 expression in cervical cancer (Chen *et al*, 2007), in which the researchers reported that 25 specimens (24.0%) showed positive immunoexpression of p-Stat3 in 104 cervical cancer specimens. This was a low occurrence rate compared with our result. This difference might be attributed to the histologic subtype or to the definition of positive expression; their data included other histological subtypes such as adenocarcinoma and microinvasive carcinoma. They also defined weak-staining specimens as having a negative expression. Their report showed that p-Stat3 expression did not significantly correlate with any clinicopathological factors such as patient's age, tumour grade, disease stage, lymph node metastasis, or histological type. However, information on clinicopathological factors seemed insufficient to draw any conclusions; approximately half the data on tumour grade, disease stage, and lymph node metastasis were not identified, and the researchers did not mention the prognosis of patients with p-Stat3 immunoexpression.

Although our result indicated that the activation of Stat3 contributed to a poor prognosis, the mechanism of constitutive activation of Stat3 has not been clarified well in cervical cancer. It is reported that the autocrine and/or paracrine system by IL-6, stimulation of EGF or Src (Zhong *et al*, 1994; Yu *et al*, 1995), mutation or overexpression of Stat3 (Bromberg, 2002; Huang *et al*, 2005) results in constitutive activation of Stat3 in other malignancies. Although a similar mechanism is considered in cervical cancer, there is no report other than that of Wei *et al* (2003) that revealed the activation of Stat3 mediated by IL-6.

Furthermore, Wei *et al* (2003) showed that the IL-6-mediated VEGF expression had been suppressed by Stat3 inhibition with a Stat3 dominant-negative mutant (DNStat3). In other malignancies, it has been suggested that the Stat3 signalling pathway regulates tumour progression by regulating downstream genes crucial to angiogenesis, proliferation, invasion, and immune evasion (Huang, 2007). Chen *et al* (2007) showed that apoptosis had been induced by the inhibition of Stat3 with DNStat3 or JSI-124, a small molecular inhibitor of the Janus-activated kinase/STAT pathway in cervical cancer. Furthermore, they showed a significant correlation of expression between p-Stat3 and antiapoptotic molecules such as BcL-xL, Mcl-1, and Survivin in immunohistochemical analysis. We also showed that the inhibition of p-Stat3 expression resulted in a downregulation of Bcl-xL and VEGF by western blotting analysis.

Recently, several inhibitors against Stat3 activation have been established that showed the induction of apoptosis or cell-cycle arrest in cancer cells, or decreased tumour volumes *in vivo* (Turkson *et al*, 2005; Xi *et al*, 2005; Duan *et al*, 2006; Hussain *et al*, 2007). Stat3 targeted therapy seems to be a promising new strategy for the treatment of cervical cancer.

In conclusion, p-Stat3 immunoexpression was found to be a potent prognostic factor of cervical cancer. Stat3 might be a target of molecular therapy in cervical cancer.

## Figures and Tables

**Table 1:** Clinical and pathological characteristics

**Characteristics**	**Number (%)**
*Age, Median*	47 (19–77)
<47	62 (49.6)
⩾47	63 (50.4)
	
*FIGO stage*	
Ib1	90 (72.0)
Ib2	11 (8.8)
IIa	11 (8.8)
IIb	13 (10.4)
	
*Tumour size*	
⩽4 cm	109 (87.2)
>4 cm	16 (12.8)
	
*Stromal invasion*	
⩽4/5	86 (68.8)
>4/5	39 (31.2)
	
*LVSI*	
Absent	60 (48.0)
Present	65 (52.0)
	
*Parametrium invasion*	
Absent	115 (92.0)
Present	10 (8.0)
	
*No. of metastatic LN*	
None	93 (74.4)
1	14 (11.2)
2	7 (5.6)
⩾3	11 (8.8)
	
*Adjuvant therapy*	
None	62 (49.6)
RT	40 (32.0)
CCRT	9 (7.2)
CT	2 (1.6)
CT+RT	12 (9.6)

CCRT=concurrent chemoradiation; CT=chemotherapy; LN=lymph nodes; LVSI=lymph vascular space invasion; RT=radiotherapy.

**Table 2:** Correlation between clinicopathologic variables and p-Stat3 expression using Fisher's exact test

**Variable**	***n***	**p-Stat3 positive (%)**	***P*-value**
*FIGO stage*			
I	101	53 (52.5)	0.006
II	24	18 (66.7)	
			
*Tumour size*			
⩽4 cm	109	56 (51.4)	0.001
>4 cm	16	15 (93.8)	
			
*Stromal invasion*			
⩽4/5	86	45 (52.3)	0.173
>4/5	39	26 (66.7)	
			
*LVSI*			
Absent	60	26 (43.3)	0.004
Present	65	45 (69.2)	
			
*Parametrium invasion*			
Absent	115	63 (54.8)	0.185
Present	10	8 (80.0)	
			
*LN metastasis*			
Absent	93	46 (49.5)	0.007
Present	32	25 (78.1)	

LN=lymph nodes; LVSI=lymph vascular space invasion.
